# Identifying significant structural factors associated with knee pain severity in patients with osteoarthritis using machine learning

**DOI:** 10.1038/s41598-024-65613-0

**Published:** 2024-06-26

**Authors:** Zhengkuan Zhao, Mingkuan Zhao, Tao Yang, Jie Li, Chao Qin, Ben Wang, Li Wang, Bing Li, Jun Liu

**Affiliations:** 1 Department of Joint, Tianjin Hospital, Tianjin, China; 2 National Elite Institute of Engineering, Chongqing University, Chongqing, China; 3 Orthopedics Department, Tianjin Hospital, Tianjin, China; 4 Tianjin Medical University, Tianjin, China; 5 School of Computer Science, Xi’an Jiaotong University, Xi’an, China

**Keywords:** Osteoarthritis, Machine learning, Knee pain severity, Convolutional neural networks, Class activation mapping, Osteoarthritis, Magnetic resonance imaging, Pain, Machine learning

## Abstract

Our main objective was to use machine learning methods to identify significant structural factors associated with pain severity in knee osteoarthritis patients. Additionally, we assessed the potential of various classes of imaging data using machine learning techniques to gauge knee pain severity. The data of semi-quantitative assessments of knee radiographs, semi-quantitative assessments of knee magnetic resonance imaging (MRI), and MRI images from 567 individuals in the Osteoarthritis Initiative (OAI) were utilized to train a series of machine learning models. Models were constructed using five machine learning methods: random forests (RF), support vector machines (SVM), logistic regression (LR), decision tree (DT), and Bayesian (Bayes). Employing tenfold cross-validation, we selected the best-performing models based on the area under the curve (AUC). The study results indicate no significant difference in performance among models using different imaging data. Subsequently, we employed a convolutional neural network (CNN) to extract features from magnetic resonance imaging (MRI), and class activation mapping (CAM) was utilized to generate saliency maps, highlighting regions associated with knee pain severity. A radiologist reviewed the images, identifying specific lesions colocalized with the CAM. The review of 421 knees revealed that effusion/synovitis (30.9%) and cartilage loss (30.6%) were the most frequent abnormalities associated with pain severity. Our study suggests cartilage loss and synovitis/effusion lesions as significant structural factors affecting pain severity in patients with knee osteoarthritis. Furthermore, our study highlights the potential of machine learning for assessing knee pain severity using radiographs.

## Introduction

Osteoarthritis (OA) stands out as one of the foremost contributors to global disability and health burdens, carrying significant personal and societal implications^1–3^. Predominantly affecting the knee joint, OA manifests primarily through the prevalent pain symptom^4,5^. Previous studies have extensively explored the interplay between structural factors and the occurrence of knee pain, structural factors, including bone marrow lesions (BMLs), cartilage damage, synovitis, and effusion, have been shown to demonstrate correlations with pain in knee osteoarthritis (KOA)^6–9^. However, few studies have considered the relationship between factors and knee pain severity^10–12^. Aligning imaging evidence with the knee pain severity and discerning the structural factors primarily linked to pain severity could inform the development of targeted, individualized treatments to alleviate symptoms and enhance patients' overall quality of life^5,7^.

KOA is commonly diagnosed using radiographs, with the Kellgren-Lawrence (KL) grades and Osteoarthritis Research Society International (OARSI) knee scores serving as widely recognized semi-quantitative assessment tools^13–15^. KL grades are commonly utilized for grading OA^16^, while OARSI scores contribute to evaluating cartilage condition^14^. Furthermore, magnetic resonance imaging (MRI) scans offer a more detailed evaluation of knee structural aspects compared to radiographs^13,17^, and the MRI-based MRI Osteoarthritis Knee Score (MOAKS) is a commonly employed semi-quantitative assessment tool known for its reliability^18,19^. These semi-quantitative scores encompass information relevant to knee pain severity. Despite their intuitiveness and convenience, semi-quantitative scores have been overlooked in previous studies concerning machine learning. Previous studies have yet to thoroughly explore the feasibility of incorporating semi-quantitative scores in model construction, with the emphasis typically placed on imaging picture information. In this study, we endeavored to construct multiple models to establish relationships between knee pain severity and semi-quantitative scores. Moreover, we assess the feasibility of using these scores in model construction, comparing them to traditional imaging picture information.

Machine learning has become a prominent tool in KOA research, with researchers developing numerous models using this method^20–22^. Researchers have demonstrated the superior performance of machine learning over traditional models in establishing correlations between imaging evidence and pain^6,14,23–25^. This shift toward machine learning underscores its potential to significantly enhance the precision and efficiency of analyses in KOA-related research. Furthermore, deep learning algorithms, particularly convolutional neural networks (CNN), have garnered attention for their remarkable ability to extract intricate visual features. These features can be effectively utilized in various applications, including disease classification, segmentation, and object detection^24,26,27^. Simultaneously, emerging image data processing methods provide an opportunity to enhance the interpretability of data features and improve model performance^28,29^. The integration of these methods has empowered researchers to analyze imaging data and assess the severity of knee osteoarthritis in a more refined and comprehensive manner, paving the way for deeper insights into the complex relationships between imaging evidence and the severity of knee osteoarthritis.

In this study, we constructed multiple machine-learning models and systematically compared imaging data to assess their potential to evaluate pain severity effectively. Subsequently, we developed a CNN to extract features from MRI images. To pinpoint the regions most closely associated with pain severity, we utilized Class Activation Mapping (CAM). These identified regions underwent thorough examination by radiologists, further validating and interpreting the findings. In summary, we have utilized various machine learning methods to explore the correlation between knee structural factors and pain severity and gain insights into the specific anatomical features contributing to pain severity, thereby contributing to a more targeted understanding and potential interventions for knee osteoarthritis.

## Methods

### Study selection

The subjects for this study were sourced from The Osteoarthritis Initiative (OAI), a multicenter, prospective, longitudinal observational study explicitly focusing on knee osteoarthritis^30^. Patients with knee osteoarthritis (KL grade ≥ 1) were selected from the baseline dataset, ensuring that they had undergone semi-quantitative assessments of knee radiographs, as well as semi-quantitative assessments of knee MRI and knee MRI. Notably, all participants enrolled in the study exhibited evidence of osteoarthritis, with at least a small osteophyte discernible on the radiographs. Following the initial pool of 600 subjects meeting these criteria, 567 subjects passed a meticulous quality check. These subjects were used for model construction and generation of CAMs. Subsequently, after screening by radiologists, we conducted a detailed examination and analysis of the CAM areas in 421 eligible patients (further details are provided below).

### MR image selection and quality check

In a subsequent study, we opted for sagittal intermediate-weighted turbo spin echo (SAG-IW-TSE) sequence images of the subjects' knees. This choice was driven by the image mode's effectiveness in capturing structural areas associated with knee pain, including BMLs, synovitis, effusion, and cartilage loss^31–34^. Following data acquisition, we undertook a comprehensive examination, and a quartile method was applied to identify and eliminate 33 abnormal images. These abnormalities encompassed instances of misalignments or the presence of foreign bodies within the slices, etc.

**Figure 1:**
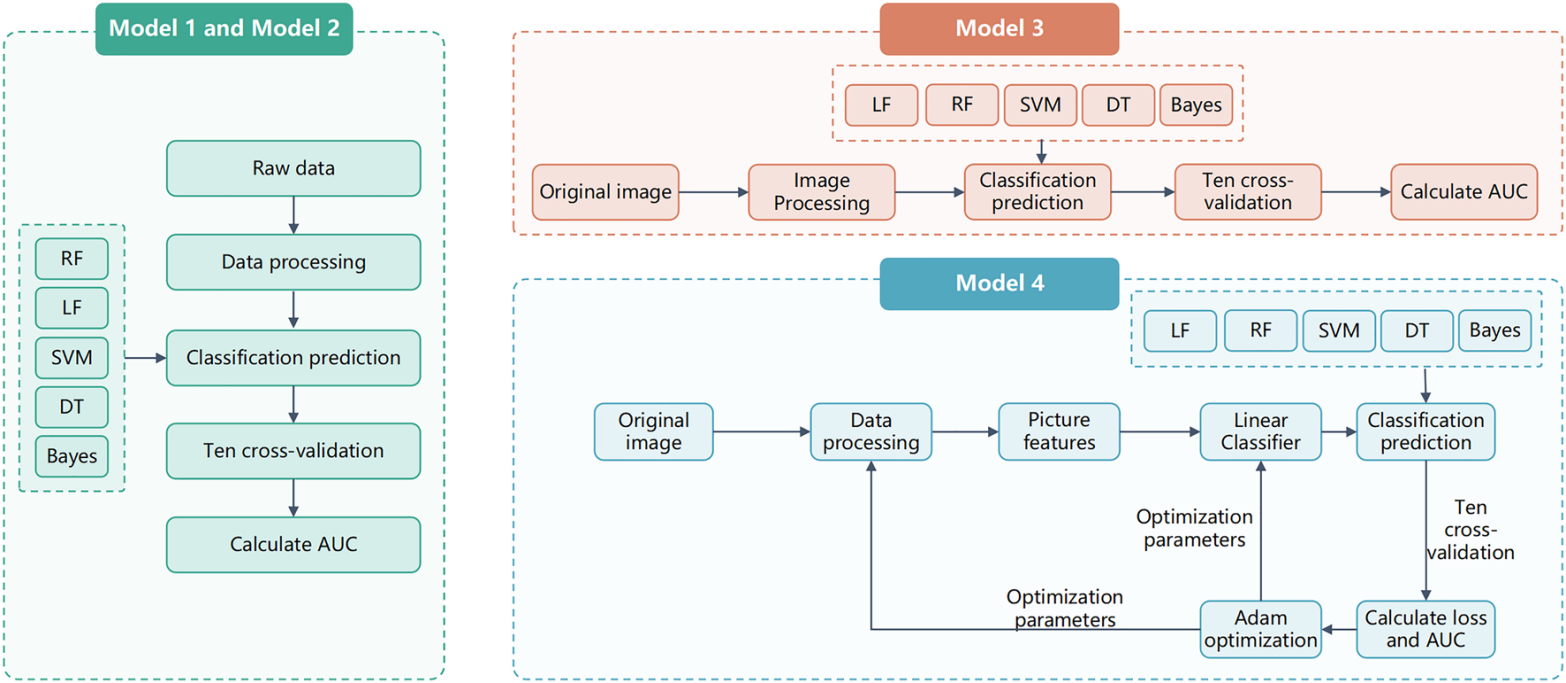
Design and construction of models. Each class of models included its sub-models.

A detailed description of the methodology employed in this study is available in the supplement S1. Here, we provide a summary (Fig. 1).

### The construction process of model 1 and model 2 (including their sub-models)

In the initial phase of our study, we developed several models to assess knee pain severity. These models were constructed based on semi-quantitative scores derived from radiographs and MRI, incorporating various structural knee lesions. The factors utilized to construct these models are outlined as follows: model 1: semi-quantitative assessment of fixed flexion knee radiographs; model 2: semi-quantitative assessment of knee MR images.

Subsequently, sub-models were constructed based on either model 1 or model 2 factors to evaluate knee pain severity further. The specific factors employed in constructing these sub-models are detailed below: model 1.1: OARSI knee scores; model 1.2: KL grades in the semi-quantitative assessment of fixed flexion knee radiographs; model 2.1: Cartilage loss in the semi-quantitative assessment of knee MR images; model 2.2: Bone marrow lesion in the semi-quantitative assessment of knee MR images; model 2.3: Meniscal damage in the semi-quantitative assessment of knee MR images; model 2.4: Osteophytes in the semi-quantitative assessment of knee MR images; model 2.5: Whole knee effusion and synovitis in the semi-quantitative assessment of knee MR images. (Effusion on the selected intermediate-weighted MR scans included effusion and synovitis; thus, effusion-synovitis were combined into a single category, as used in MOAKS). For a more detailed description of the factors employed in these models, additional information is available in the supplement S1 accompanying this study.

In the development of model 1 and model 2, as well as other models derived from these base models, we employed the K-nearest neighbors (KNN) imputation method to handle missing data. To ensure robustness and mitigate any potential influence of data order on the results, we randomized the order of the data to achieve greater robustness.

### The construction process of model 3 and model 4

Subsequently, we used neural networks and constructed models 3 and 4 to assess knee pain severity. The factors used to construct these models are model 3: knee MR images (four sections of SAG-IW-TSE images) and model 4: knee MR images after feature extraction (four sections of SAG-IW-TSE images).

For model 3, we applied the normalization method to preprocess the data, transforming the two-dimensional images into one-dimensional vectors. Again, we randomized the data to improve the results' validity. For model 4, we used the image features extracted from the images (see below) to generate a dataset, which was then processed using the same method as model 3.

### Constructed models

The construction method of each type of data was consistent. We utilized grid search to tune the hyperparameters and constructed models using five different methods: random forests (RF), support vector machines (SVM), logistic regression (LR), decision tree (DT), and Bayesian (Bayes). To maintain consistency across the models, we divided the data into training and testing sets (with randomized indices) using a consistent ratio of 9:1. Additionally, tenfold cross-validation was employed for all models. Ultimately, we selected models with the highest performance. This approach ensures that the chosen models exhibit reliability in assessing knee pain severity across various subsets of the dataset.

### Results evaluation and models performance

The evaluation of OA-induced knee pain severity utilized the Western Ontario and McMaster Universities Osteoarthritis (WOMAC) pain scale, which ranges from 0 to 20. This scale is derived from a multi-item survey assessing pain experienced during various activities. Knee pain was categorized into four groups based on the WOMAC pain score: no pain (score = 0), mild pain (0 < score < 4), moderate pain (score ≥ 4), and severe pain (score ≥ 8)^13,35,36^.

The area under the curve (AUC) of the receiver operating characteristic (ROC) curves was utilized as a metric for the performance of the models. When comparing models, we ensured that all other conditions and methods remained consistent. Models with higher performance typically indicated stronger correlations and were thus deemed more effective in assessing knee pain severity. All models were constructed using Python 3.9.

### Development of the CNN

**Figure 2:**
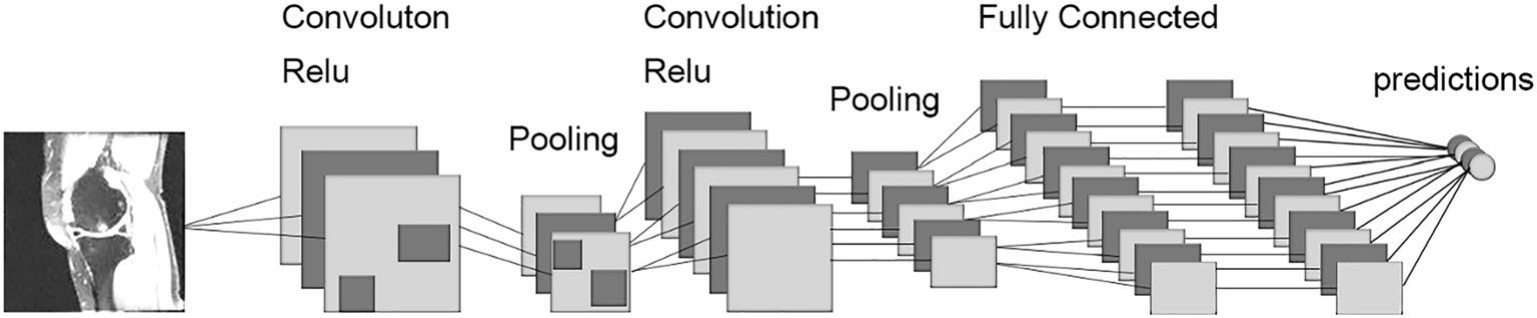
Convolutional Siamese network architecture.

When constructing the neural network models, we incorporated batch normalization and Rectified Linear Unit (ReLU) as the normalization and non-linear function layers, respectively. Max pooling served as the pooling method, and the architecture included five convolutional layers and three pooling layers stacked together. The output from these layers was connected to the output of three fully connected layers. The input was a two-dimensional image, and the output was a vector with the same length as the number of labels representing knee pain severity. The training process utilized the AdamW optimizer and the cross-entropy loss function. Each iteration involved gradient clearing, model forward propagation, loss function backpropagation, and gradient optimization. The classification accuracy of the training set was recorded in each iteration. Additionally, forward propagation was conducted on the validation set, and the classification accuracy on the validation set was recorded. If the correct rate of the validation set in a given round exceeded the historical correct rate, the model was stored in a pkl file. Figure 2 illustrates the schematic of the CNN.

### Generation of the CAMs

In our CNN model, we conducted global average pooling (GAP) on the final feature map to compute the mean value of each channel, which was then mapped to the class score through a Fully Connected (FC) layer. The argmax was determined, and the gradient of the last feature map was computed. This gradient was subsequently visualized on the original image, generating the CAM. During this visualization process, a heatmap intensity factor of 0.4 was set to achieve the desired CAM. We concatenated the CAMs obtained from the four sections of each patient's images to present the final results. This approach allows for a comprehensive visualization of the regions within the knee images that significantly contribute to the model's assessment of knee pain severity.

### Radiologist review the CAMs

The musculoskeletal radiologist, with significant expertise in knee MRI interpretation manually reviewed images and excluded those with unsatisfactory feature extraction or MRI quality. To streamline further analysis, the radiologist specifically selected subjects whose CAM regions were primarily focused on a single lesion within the knee. This targeted selection aimed to ensure that the analysis concentrated on cases where the model's attention was distinctly directed toward a specific anatomical feature. Subsequently, we performed a statistical analysis on this refined set of subjects. This approach ensures that the results and conclusions drawn from the analysis are based on a subset of subjects where the model's attention is mainly focused and interpretable.

### Informed consent

Because de-identified data was sourced from the publicly available Osteoarthritis Initiative (OAI) database (https://oai.nih.gov), informed consent was unnecessary.

## Results

### Participant characteristics

In this study, 567 subjects participated in constructing the models, with a mean age of 61.4 ± 8.9 years and a mean body mass index of 30.8 ± 4.8 kg/m^2^. Table 1 details the characteristics of the participants. Among these 567 participants, a subset of 421 participants was included in the further analysis focusing on specific lesions colocalized with CAM regions associated with knee pain severity. The demographic characteristics of the 421 participants can be found in the S1 Table of the Supplementary Material.

**Table 1:** Demographic and baseline characteristics.

Subjects	No pain	Mild pain	Moderate pain	Severe pain	Combine
Age	n = 216	n = 199	n = 100	n = 52	n = 567
Mean (SD)	62.2 (9.0)	61.2 (8.7)	60.9 (9.2)	60.1 (8.2)	61.4 (8.9)
Min, max	45–79	45–78	45–79	45–74	45–79
Race	n = 216	n = 199	n = 100	n = 52	n = 567
White	179 (82.8%)	162 (81.4%)	73 (73.0%)	32 (61.5%)	446 (78.7%)
Black	30 (13.9%)	33 (16.6%)	24 (24.0%)	18 (34.6%)	105 (18.5%)
Asian or other	7 (0.03%)	4 (2%)	3 (3%)	2 (3.9%)	16 (2.8%)
BMI (m/kg^2^)	n = 216	n = 199	n = 99	n = 52	n = 566
Mean (SD)	29.8 (4.6)	31.1 (4.5)	31.3 (5.0)	32.4 (5.3)	30.8 (4.8)
Min, max	18.6 to 42.5	20 to 43.9	20.6 to 46.7	18.8 to 46	18.6 to 46.7
Use of NSAIDs at baseline	n = 216	n = 199	n = 100	n = 52	n = 567
Yes	50 (23.1%)	48 (24.1%)	32 (32.0%)	15 (28.9%)	145 (25.6%)
No	166 (76.9%)	151 (75.9%)	68 (68.0%)	37 (71.1%)	422 (74.4%)
Comorbidity	n = 216	n = 198	n = 98	n = 51	n = 563
Yes	50 (23.1%)	48 (24.2%)	33 (33.7%)	21 (41.2%)	155 (27.5%)
No	166 (76.9%)	150 (75.8%)	65 (66.3%)	30 (58.8%)	408 (72.5%)
Risk factor	n = 215	n = 199	n = 100	n = 52	n = 566
Yes	161 (74.9%)	147 (73.9%)	72 (72%)	40 (76.9%)	420 (74.2%)
No	54 (25.1%)	52 (26.1%)	28 (28%)	12 (23.1%)	146 (25.8%)
Gender	n = 216	n = 199	n = 100	n = 52	n = 567
Male	94 (43.5%)	78 (39.2%)	45 (45.0%)	15 (28.8%)	232 (40.9%)
Female	122 (56.4%)	121 (60.8%)	55 (55.0%)	37 (71.2%)	335 (59.1%)

*BMI denotes body mass index, NSAIDS denotes nonsteroidal anti-inflammatory drugs.

### The performance of models

In evaluating model 1 and model 2, along with their sub-models, the svm approach yielded the highest performance, except for model 2.1, which achieved the optimal performance with the LR approach. Specifically, model 1 achieved an optimal AUC of 0.680, while model 1.1 and model 1.2 achieved optimal AUCs of 0.677 and 0.678, respectively. model 2 achieved optimal AUC of 0.671, and its submodels (model 2.1, model 2.2, model 2.3, model 2.4, and model 2.5) achieved optimal AUCs of 0.681, 0.681, 0.672, 0.682, and 0.671, respectively. Models 3 and 4, employing the RF approach, demonstrated superior performance with optimal AUCs of 0.690 and 0.698, respectively. Refer to S2 Table of the Supplementary Material for detailed performance results.

**Figure 3:**
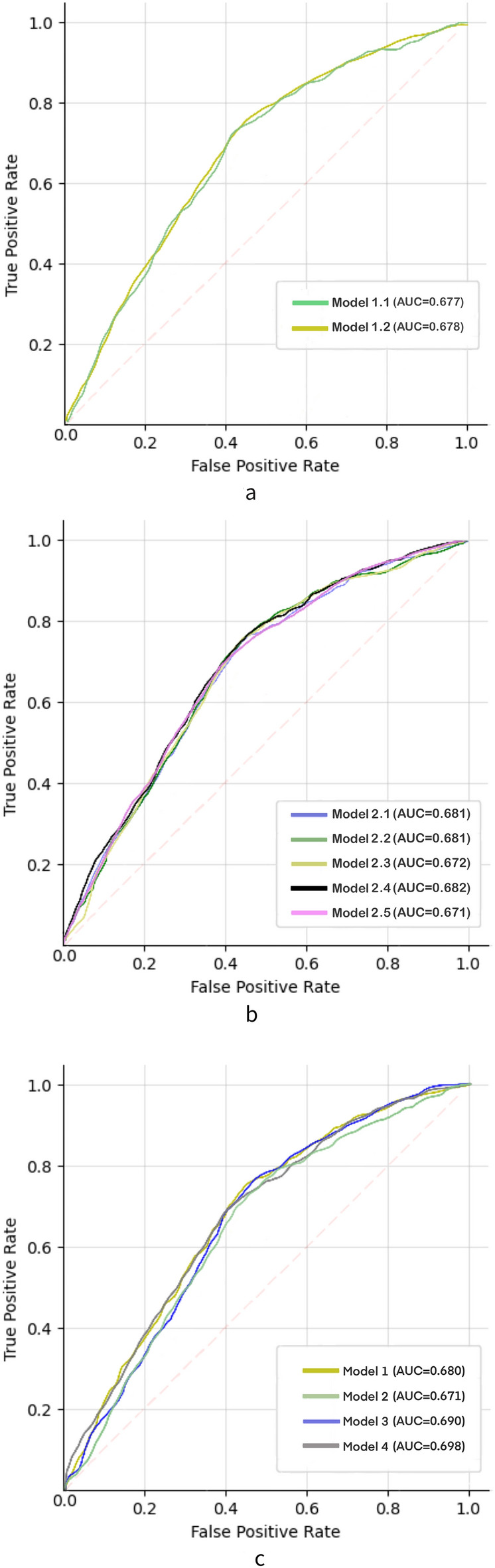
Receiver operating characteristic (ROC) curves were generated to compare the performance of different models. (**a**) Comparison of area under the curve (AUC) among subgroups of model 1. (**b**) Comparison of AUC among subgroups of model 2. (**c**) Comparison of AUC among model 1, model 2, model 3, and model 4.

The results of the comparison between the model are presented in Fig. 3a–c, revealing that the differences in performance among the various models are not substantial. Model 4 slightly outperforms over model 3, and similarly, model 1 slightly outperforms model 2. Additionally, minimal differences in performance were observed between the sub-groups of model 1 and model 2.

### Feature extraction and radiologist identification

**Figure 4:**
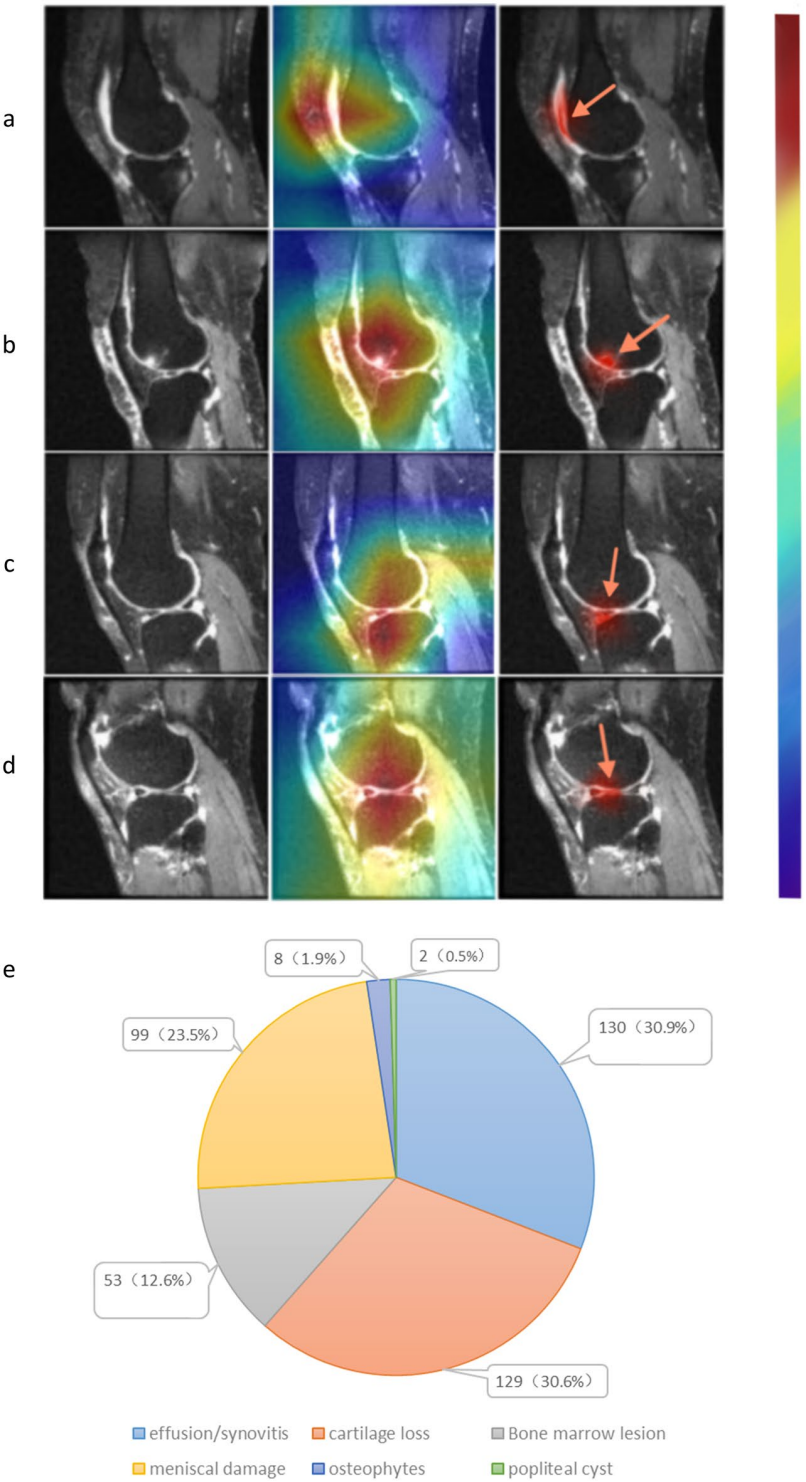
The left column showed the original sagittal MRI, while the middle column showed the CAMs of the selected subjects. The right column showed the specific lesion identified by the radiologist. In detail, effusion/synovitis (**a**), meniscus damage (**b**), bone marrow lesions (**c**), and cartilage loss (**d**) were the lesions present within the CAMs. (**e**) Displays the corresponding number of each lesion in these cases.

Utilizing CAMs derived from features extracted in the final convolutional layer of the neural network enabled us to examine regions strongly correlated with knee pain severity. Following a thorough review by a radiologist, lesions encompassing effusion-synovitis, cartilage loss, meniscal damage, BMLS, and popliteal cysts were identified in 421 cases (Fig. 4a–d). The most pertinent structural abnorm + alities associated with knee pain severity were effusion-synovitis and cartilage loss, prevalent in 30.9% (130) and 30.6% (129) of subjects, respectively. Meniscal damage was evident in 23.5% (99) of subjects, while BMLS and osteophytes were observed in 12.6% (53) and 1.9% (8) of subjects, respectively. Popliteal cysts were identified in only 0.5% (2) of subjects (Fig. 4e). These results underscored effusion-synovitis and cartilage loss as the most frequently encountered abnormalities in cases of knee pain severity. Instances of meniscal damage outnumbered those of BMLS, whereas osteophytes and popliteal cysts were relatively rare, with only a few cases.

## Discussion

We developed models to evaluate knee pain severity in patients with KOA using diverse datasets. In general, the MRI semi-quantitative score provided more detailed knee features than the radiographic semi-quantitative score, which cannot directly capture pain-associated features such as BMLS and synovitis^6–8^. Surprisingly, our results indicated that model 1 slightly outperformed model 2, implying that radiographs may hold the potential to be as effective as MRIs in assessing pain severity through machine learning. This counterintuitive finding challenges the assumption that the richness of detail in MRI features would inherently lead to superior pain severity assessments. It suggests that the specific information provided by radiographs, though less detailed, may still contribute meaningfully to the evaluation of knee pain severity in the context of machine learning. Our results align with Neogi T et al., who considered individual radiographic features strongly correlated with knee pain^37^. Similarly, our study suggested that the correlation between radiographs and knee pain severity may be underestimated.

Additionally, our analysis revealed that model 3 does not significantly differ from model 2. This observation suggests that leveraging the MOAKS effectively enables the extraction of information from MRI for pain severity assessment. We adopted an intuitive approach to compare the performance of knee pain severity assessment between semi-quantitative assessment of MRI and direct evaluation of MRI images, thus demonstrating the reliability of the semi-quantitative assessment method in gauging pain severity.

For further research, we developed several sub-models based on model 1 and model 2. We systematically analyzed the structural factors associated with knee pain severity using the same samples for modeling. In our study, both model 1.1 and model 1.2 exhibited similar performance, suggesting that there may be little difference in the potential of the KL classification and the OARSI knee score in assessing the severity of knee pain using a machine learning approach. Furthermore, subgroup comparisons within model 2 revealed specific lesions that were correlated with pain severity. Models constructed for different types of lesion areas have shown assessment potential. Our findings align with prior studies by L. Torres et al., where cartilage loss, bone marrow lesions, effusion-synovitis, and meniscal damage were all associated with knee pain severity^38^. Additionally, our study revealed that osteophytes were also associated with pain severity, which is consistent with Sayre et al.'s findings that consistently demonstrated an association between osteophytes and pain severity, both cross-sectionally and longitudinally^39^.

Due to the absence of significant differences in performance among the subgroups of model 2. We could not directly ascertain which lesion factors had a more pronounced effect on pain severity. To gain a deeper understanding of the lesion factors substantially impacting pain severity, we constructed a CNN, followed by the generation of CAMs. Model 4 exhibited slightly superior performance compared to model 3, indicating successful extraction of critical information related to pain severity. To pinpoint the regions of interest, the radiologist identified the CAMs. We found that effusion-synovitis lesions and cartilage loss were more prevalent than other lesions, and meniscal damage was more common than BMLs. It indicated that cartilage loss and effusion-synovitis lesions are significant structural factors influencing pain severity in KOA patients.

It is crucial to note that our study focused on pain severity rather than pain itself. The relationship between these lesions is intricate, such as the interaction between cartilage loss and synovitis^40,41^. Although our post-processing results suggested that effusion-synovitis and cartilage loss were the most common abnormalities associated with pain severity, we did not imply a causal pain mechanism. Osteophytes were not discussed due to difficulty in identification on sagittal MRI, and popliteal cysts were not further discussed due to their lower prevalence.

Our study represents the first attempt to integrate semi-quantitative knee joint scoring into the model and conduct comparative analyses. We employed an intuitive approach to assess the performance of knee pain severity evaluation, comparing the semi-quantitative assessment of MRI with the direct evaluation of MRI images. Additionally, we assessed the reliability of the semi-quantitative assessment method for gauging pain severity. Finally, we introduced a novel method of manual annotation interpretation, endeavoring to explain the CAM regions obtained from our CNN model. This effort provides a new perspective on the applicability of neural network interpretability in this field and confirms its feasibility.

This study comes with certain limitations. Despite employing tenfold cross-validation, there might be minor fluctuations in the performance of our models. However, our emphasis was on model comparison rather than assessing their specific performance. Additionally, although sagittal MRI proves effective in capturing crucial structural regions linked to knee pain, relying solely on this imaging modality for pain severity assessment could impact both the model's performance and the radiologists' accuracy in identifying specific lesions. Notably, we could not quantify the significance of the correlation between these identified lesions and pain severity. Further studies are warranted to determine the magnitude of the association between these structural factors and pain severity.

In conclusion, our study employed machine learning approaches to confirm the potential of radiographs in assessing knee pain severity. Our findings reveal associations between knee pain severity and structural factors, including cartilage loss, bone marrow lesions, osteophytes, effusion-synovitis, and meniscal damage. Particularly, cartilage loss and effusion-synovitis lesions emerged as substantial structural factors significantly influencing pain severity in KOA patients. These results hold promise for clinical guidance in targeting the treatment or relief of pain caused by OA, and they present novel research ideas for the application of machine learning in advancing OA-related research.

### Supplementary Information

Supplementary Information.

## Data Availability

Our research data is derived from the publicly available Osteoarthritis Initiative (OAI) database (https://oai.nih.gov), and all the data is available in this database.

## Code availability

The codes utilized in our models can be found at: https://github.com/Harry-Miral/ML-python.
