# Supplementary material for: Identifying significant structural factors associated with knee pain severity in patients with osteoarthritis using machine learning
Source: Sci Rep. 2024 Jun 26;14:14705. doi: 10.1038/s41598-024-65613-0 (PMC11208546; doi:10.1038/s41598-024-65613-0)
Supplement: Supplementary file 1 — Supplementary Information. [file 41598_2024_65613_MOESM1_ESM.docx]

**Supplemental information**

**Models codes**

The codes utilized in our models can be found at https://github.com/Harry-Miral/ML-python.

**Assessment factors of models**

Several classes of models were constructed to assess knee pain severity. It was assumed that all lesions were related to pain severity. The assessment factors used to construct these models are as follows: Model 1: Semi-quantitative assessment of fixed flexion knee radiographs (OARSI knee scores including joint space width (JSW) and KL grades (osteophytes, attrition, cysts, etc.); Model 2: Semi-quantitative assessment of knee MR images (MOAKS and other supplementary items (cartilage integrity, osteophytes, cysts, meniscal integrity, effusion, ligament integrity, etc.); Model 3: Knee MR images (four sections of SAG-IW-TSE images); Model 4: Knee MR images after feature extraction (four sections of SAG-IW-TSE images).

Submodels were constructed based on Model 1 or Model 2 to assess knee pain severity. These assessment factors selected directly from the database.The assessment factors used to construct these sub-models are as follows:

**Model 1.1: OARSI knee scores (including JSW in the semi-quantitative assessment of fixed flexion knee radiographs):**osteophytes (OARSI grades 0-3) femur medial compartment;sclerosis (OARSI grades 0-3) femur medial compartment;joint space narrowing (OARSI grades 0-3) medial compartment;osteophytes (OARSI grades 0-3) tibia medial compartment;sclerosis (OARSI grades 0-3) tibia medial compartment;attrition (OARSI grades 0-3) tibia medial compartment;osteophytes (OARSI grades 0-3) femur lateral compartment;sclerosis (OARSI grades 0-3) femur lateral compartment;joint space narrowing (OARSI grades 0-3) lateral compartment;osteophytes (OARSI grades 0-3) tibia lateral compartment;sclerosis (OARSI grades 0-3) tibia lateral compartment;attrition (OARSI grades 0-3) tibia lateral compartment

**Model 1.2: KL grades (including osteophytes, attrition, cysts, etc.) in the semi-quantitative assessment of fixed flexion knee radiographs:**cysts (Grades 0-1) femur medial compartment; chondrocalcinosis (Grades 0-1) medial compartment;cysts (Grades 0-1) tibia medial compartment; Kellgren and Lawrence (grades 0-4);cysts (Grades 0-1) femur lateral compartment; chondrocalcinosis (Grades 0-1) lateral compartment;cysts (Grades 0-1) tibia lateral compartment

**Model 2.1: Cartilage loss in the semi-quantitative assessment of knee MR images:** MOAKS: cartilage morphology - patella medial; cartilage morphology - patella lateral; cartilage morphology - femur medial anterior (trochlear); cartilage morphology - femur lateral anterior (trochlear); cartilage morphology - femur medial posterior ; cartilage morphology - femur lateral posterior ; cartilage morphology - femur medial central ; cartilage morphology - femur lateral central ; cartilage morphology - tibia medial anterior ; cartilage morphology - tibia lateral anterior ; cartilage morphology - tibia medial central ; cartilage morphology - tibia lateral central ; cartilage morphology - tibia medial posterior ; cartilage morphology - tibia lateral posterior

**Model 2.2: Bone marrow lesion in the semi-quantitative assessment of knee MR images:** MOAKS:BML size - femur medial anterior (trochlear);BML (% lesion that is edema) - femur medial anterior (trochlear);number of BML lesions - femur medial anterior (trochlear);BML size - femur

lateral anterior (trochlear) ;BML (% lesion that is edema) - femur lateral anterior (trochlear) ;number of BML lesions - femur lateral anterior (trochlear) ;BML size - femur medial central ;BML (% lesion

that is edema) - femur medial central ;number of BML lesions - femur medial central ;BML size - femur lateral central ;BML (% lesion that is edema) - femur lateral central ; number of BML

lesions - femur lateral central ;BML size - femur medial posterior ;BML (% lesion that is edema) - femur medial posterior; number of BML lesions - femur medial posterior ;BML size - femur

lateral posterior ;BML (% lesion that is edema) - femur lateral posterior tggpv ;number of BML

lesions - femur lateral posterior ;BML size - tibia sub-spinous ;BML (% lesion that is edema) - tibia sub-spinous ;number of BML lesions - tibia sub-spinous ;BML size - tibia medial anterior ;BML (% lesion that is edema) - tibia medial anterior ;number of BML lesions - tibia medial anterior;BML size - tibia lateral anterior ;BML (% lesion that is edema) - tibia lateral anterior ;number of BML lesions - tibia lateral anterior ;BML size - tibia medial central ;BML (% lesion that is edema) - tibia medial central ;number of BML lesions - tibia medial central ;BML size - tibia lateral central ;BML (% lesion

that is edema) - tibia lateral central ;number of BML lesions - tibia lateral central ;BML size - tibia

medial posterior ;BML (% lesion that is edema) - tibia medial posterior ;number of BML lesions - tibia medial posterior ;BML size - tibia lateral posterior ;BML (% lesion that is edema) - tibia lateral posterior ;number of BML lesions - tibia lateral posterior ;BML size - patella medial ;BML (% lesion

that is edema) - patella medial ;number of BML lesions - patella medial ;BML size - patella lateral

;BML (% lesion that is edema) - patella lateral ;number of BML lesions - patella lateral

**Model 2.3: Meniscal damage in the semi-quantitative assessment of knee MR images:** MOAKS:medial meniscal morphology - anterior horn ;lateral meniscal morphology - anterior horn ;medial meniscal morphology - body ;lateral meniscal morphology - body ;medial meniscal morphology - posterior horn ;lateral meniscal morphology - posterior horn ;medial meniscal hypertrophy - anterior horn ;lateral meniscal hypertrophy - anterior horn ;medial meniscal hypertrophy - body ;lateral meniscal hypertrophy - body ;medial meniscal hypertrophy - posterior horn ;lateral meniscal hypertrophy - posterior horn ;medial meniscal signal abnormality - anterior horn ;lateral meniscal signal abnormality - anterior horn ;medial meniscal signal abnormality - body ;lateral meniscal signal abnormality - body ;medial meniscal signal abnormality - posterior horn ;lateral meniscal signal abnormality - posterior horn ;medial meniscal extrusion - medially ;medial meniscal extrusion - anteriorly ;lateral meniscal extrusion - anteriorly;lateral meniscal extrusion-laterally ;medial meniscal morphology - posterior root tear ;lateral meniscal morphology - posterior root tear.

**Model 2.4: Osteophytes in the semi-quantitative assessment of knee MR images:**

MOAKS:osteophyte size - patella superior ;osteophyte size - patella inferior ;osteophyte size - patella medial ;osteophyte size - patella lateral ;osteophyte size - femur medial anterior (trochlear) ;osteophyte

size - femur lateral anterior (trochlear) ;osteophyte size - femur medial posterior ;osteophyte size - femur lateral posterior ;osteophyte size - femur medial central ;osteophyte size - femur lateral central

;osteophyte size - tibia medial ;osteophyte size - tibial lateral

**Model 2.5: Whole knee effusion and synovitis in the semi-quantitative assessment of knee MR images** (Effusion on the selected intermediate-weighted MR scans included effusion and synovitis; thus, effusion-synovitis were combined into a single category, as used in MOAKS)**:** MOAKS: inter-condylar synovitis ;whole knee effusion

**Detailed descriptions of the methodology**

**Importing Third-Party Libraries**

The implementation of the machine learning method involved importing several third-party libraries. Commonly used libraries such as pandas, numpy, os, and PIL were imported for image file import and matrix calculation. Furthermore, matplotlib was used for drawing result graphs, and sklearn was used to employ pre-packaged machine learning classifiers.

**Data Reading**

The image file was read ,and the type and original size of the image were output. Using the resize function, the size of the image was changed. The figs folder was looped through to discretize the images and store them in a three-dimensional tensor data_fig. Additionally, the excel table was read and the table data, that is, image label data, was stored in the variable data.

**Data Preprocessing**

Preprocessing was performed on the data by normalizing the 3D tensor data_fig containing image information from 0 to 255. The reshape function was then used to convert the 2D image into a 1D vector. The processed data_fig was then concatenated with the label data, resulting in a one-to-one correspondence between image vectors and labels. The labels were then rounded, and the order of image-vector-label pairs was randomly shuffled. Outliers were subsequently removed from the data pairs, and the dataset was divided into ten equal parts. These parts were stored in a dictionary, and the scipy.io library was used to store the image vectors and corresponding image classes in .mat format.

**Convolutional Neural Network (CNN) Training and Validation**

The CNN training and validation were performed cyclically on the ten data parts. During training, nine pieces of data were used as the training set, and one piece of data was used as the test set. In this process, training hyperparameters such as learning rate, number of training epochs, batch size, etc., were first defined. The MyDataset class was then built to read the dataset. The Load function in load_data.py was called to read the image vector in the .mat file and restore it to a two-dimensional matrix. An iterator was used in the MyDataset class to iterate over the output 2D image label pairs. Next, a CNN network was constructed, with batchnorm and relu as normalization, non-linear function layers, and maxpool as the pooling method. Five convolutional layers and three pooling layers were stacked together, with the last three fully connected layers connected to the output. The input was a 2D image, and the output was a vector of the same length as the number of labels. AdamW optimizer and cross-entropy loss function were used for training. In each iteration, gradient removal, model forward propagation, loss function backpropagation, gradient optimization, and the classification accuracy of the training set were recorded. Next, the forward propagation of the validation set was performed, and the classification accuracy of the validation set was recorded. The model was then stored in a pkl file if the correct rate of this round of the validation set was greater than the historical correct rate.

**Classification prediction was performed using various machine learning algorithms.**

Specifically, five functions, namely CrossValidationForRF, CrossValidationForSVM, CrossValidationForLR, CrossValidationForTRE, and CrossValidationForBYS, were defined to predict the training and test sets using random forest classifier, support vector machine classifier, logistic regression model, decision tree model grid search method, and K nearest neighbor model Bayesian search method, respectively. In the CrossValidationForRF function, hyperparameters were sequentially defined, and the results were computed iteratively using the OneVsRestClassifier's classifier-building strategy. The model was then fit and predicted, and the Receiver Operating Characteristic (ROC) and Area Under the Curve (AUC) were calculated. Similarly, in the CrossValidationForSVM function, the hyperparameter optimization method of grid search GridSearchCV was defined, and the results of model fitting, model prediction, ROC, and AUC were calculated using the OneVsRestClassifier's classifier construction strategy.

The CrossValidationForLR function defined hyperparameters in turn and used the hyperparameter optimization method of grid search GridSearchCV. The classifier construction strategy of OneVsRestClassifier was iteratively adopted to build a classifier with the same number of labels, and model fitting, model prediction, calculation of ROC and AUC were performed. The CrossValidationForTRE and CrossValidationForBYS functions followed similar logic.After defining the five functions, the ten parts of the dataset were traversed, and the data were read and filtered in turn. The data were trained and predicted using these five functions, and the training and testing AUC results and optimal hyperparameters were stored in a list. It should be noted that when calculating AUC, the predicted labels were converted to one-hot form.Next, the one-hot form of the predicted output results from the five functions was used to calculate the false positive rate (FPR) and the true positive rate (TPR). These rates were printed out, and the ROC curve was drawn using the matplotlib library. The AUCs of the five classification methods were compared, and the best classification method was found to be the RF Random Forest classification method, with its mean and maximum and minimum values plotted and displayed.

**S1 Table**

| **S1 Table Demographic characteristics of subjects after radiologist review** | | | | | |
| --- | --- | --- | --- | --- | --- |
| Subjects | no pain | mild pain | moderate pain | severe pain | combine |
| Age | n=160 | n=148 | n=75 | n=38 | n=421 |
| Mean(SD) | 61.8（9.1） | 60.7(8.5) | 61.0(9.0) | 61.2(9.1) | 61.2(8.9) |
| Min,Max | 45 to 79 | 45 to 79 | 45 to 79 | 45 to 79 | 45 to 79 |
| Race | n=160 | n=148 | n=75 | n=38 | n=421 |
| White | 133(83.1%) | 121(81.7%) | 53 (70.6%) | 25(65.7%) | 332(78.8%) |
| Black | 27(16.8%) | 27(18.2%) | 22(29.3%) | 13(34.2%) | 89(21.1%) |
| BMI(m/kg²) | n=160 | n=148 | n=75 | n=38 | n=421 |
| Mean(SD) | 30.1(4.9) | 31.0(4.6) | 31.7(4.7) | 32.1(5.5) | 30.9(4.8) |
| Min,Max | 18.6 to 42.5 | 22.4 to 43.9 | 23.2 to 46.7 | 18.8 to 46 | 18.6 to 46.7 |
| Use of NSAIDs at Baseline | n=160 | n=148 | n=75 | n=38 | n=421 |
| Yes | 14(8.7%) | 12(8.1%) | 14(18.6%) | 8(21.0%) | 48(11.4%) |
| No | 146(91.2%) | 136(91.8%) | 61(81.3%) | 30(78.9%) | 373(88.5%) |
| Comorbidity | n=160 | n=148 | n=75 | n=38 | n=418 |
| Yes | 39(24.3%) | 33(22.2%) | 22(29.3%) | 17(44.7%) | 111(26.3%) |
| No | 121(75.6%) | 115(77.7%) | 51(68.0%) | 20(52.6%) | 307(72.9%) |
| Risk Factor | n=160 | n=148 | n=75 | n=38 | n=420 |
| Yes | 120(75.0%) | 109(73.6%) | 55(73.3%) | 26(68.4%) | 310(73.6%) |
| No | 39(24.3%) | 39(26.3%) | 20(26.6%) | 12(31.5%) | 110(26.1%) |
| Gender | n=160 | n=148 | n=75 | n=38 | n=421 |
| male | 64(40.0%) | 59(39.8%) | 34(45.3%) | 12(31.5%) | 169(40.1%) |
| female | 96(60.0%) | 89(60.1%) | 41(54.6%) | 26(68.4%) | 252(59.8%) |

*BMI denotes body mass index, NSAIDS denotes nonsteroidal anti-inflammatory drugs

**S2 Table: The specific performance of each model.**

|  | RF | SVM | LR | DT | Bayes |
| --- | --- | --- | --- | --- | --- |
| Model 1 | 0.664 | 0.680 | 0.677 | 0.648 | 0.580 |
| Model 1.1 | 0.661 | 0.675 | 0.677 | 0.665 | 0.619 |
| Model 1.2 | 0.664 | 0.678 | 0.669 | 0.636 | 0.601 |
| Model 2 | 0.645 | 0.671 | 0.649 | 0.655 | 0.580 |
| Model 2.1 | 0.659 | 0.681 | 0.659 | 0.673 | 0.589 |
| Model 2.2 | 0.661 | 0.681 | 0.663 | 0.674 | 0.587 |
| Model 2.3 | 0.657 | 0.672 | 0.664 | 0.668 | 0.581 |
| Model 2.4 | 0.668 | 0.682 | 0.666 | 0.653 | 0.630 |
| Model 2.5 | 0.657 | 0.671 | 0.667 | 0.667 | 0.600 |
| Model 3 | 0.690 | 0.667 | 0.652 | 0.667 | 0.560 |
| Model 4 | 0.698 | 0.640 | 0.634 | 0.599 | 0.576 |
